# Use of Arsenic in Ascities

**Published:** 1845-10

**Authors:** 


*Use of Arsenic in Ascites.* By Dr. Cormack.—This gentleman believes that when ascites depends upon hepatic or splenic congestion, much benefit will be obtained from the administration of small doses of arsenic. He recommends that the dose should not at first exceed the 16th or 20th part of a grain; that this should be taken in not less than four ounces of water, and with an empty stomach. The dose should be repeated four or six times—not seldomer—in 24 hours. In skin diseases, intermittent fevers, enlarged spleen from intermittent fever, and, indeed, in every case in which arsenic is known to have specific effects, Dr. C. recommends that it should be given in the method above mentioned, and advises that all other drugs should, if at all possible, be suspended during its administration. In old cases of enlarged spleen and liver, the cure will be hastened by the use of a succession of blisters.—*Lond. and Edin. Monthly Jour. of Med. Sci.—Ibid.*

